# The Effects of Sarcopenia on Overall Survival and Postoperative Complications of Patients Undergoing Hepatic Resection for Primary or Metastatic Liver Cancer: A Systematic Review and Meta-Analysis

**DOI:** 10.3390/jcm13133869

**Published:** 2024-06-30

**Authors:** Alexandros Giakoustidis, Menelaos Papakonstantinou, Paraskevi Chatzikomnitsa, Areti Danai Gkaitatzi, Petros Bangeas, Panagiotis Dimitrios Loufopoulos, Eleni Louri, Athanasia Myriskou, Ioannis Moschos, Diomidis Antoniadis, Dimitrios Giakoustidis, Vasileios N. Papadopoulos

**Affiliations:** 1 A’ Department of Surgery, General Hospital Papageorgiou, School of Medicine, Faculty of Medical Sciences, Aristotle University of Thessaloniki, 56429 Thessaloniki, Greece; menelaospap.md@gmail.com (M.P.); voula.hatzikomnitsa@gmail.com (P.C.); aretidanaegtz24@gmail.com (A.D.G.); pbangeas@gmail.com (P.B.); loufopoulosp@gmail.com (P.D.L.); elenilouri@gmail.com (E.L.); myriskou@gmail.com (A.M.); dgiakoustidis@gmail.com (D.G.); papadvas@auth.gr (V.N.P.); 2 International Hellenic University, 56429 Thessaloniki, Greece; gutgutgut2011@gmail.com; 3 School of Medicine, Aristotle University of Thessaloniki, 56429 Thessaloniki, Greece; dio_psych@yahoo.gr

**Keywords:** sarcopenia, liver resection, overall survival, complications

## Abstract

**Background:** Colorectal cancer is the third most common cancer worldwide, and 20–30% of patients will develop liver metastases (CRLM) during their lifetime. Hepatocellular carcinoma (HCC) is also one of the most common cancers worldwide with increasing incidence. Hepatic resection represents the most effective treatment approach for both CRLM and HCC. Recently, sarcopenia has gained popularity as a prognostic index in order to assess the perioperative risk of hepatectomies. The aim of this study is to assess the effects of sarcopenia on the overall survival (OS), complication rates and mortality of patients undergoing liver resections for HCC or CRLM. **Methods:** A systematic literature search was performed for studies including patients undergoing hepatectomy for HCC or CRLM, and a meta-analysis of the data was performed. **Results:** Sarcopenic patients had a significantly lower 5-year OS compared to non-sarcopenic patients (43.8% vs. 63.6%, respectively; *p* < 0.01) and a significantly higher complication rate (35.4% vs. 23.1%, respectively; *p* = 0.002). Finally, no statistical correlation was found in mortality between sarcopenic and non-sarcopenic patients (*p* > 0.1). **Conclusions:** Sarcopenia was significantly associated with decreased 5-year OS and increased morbidity, but no difference was found with regard to postoperative mortality.

## 1. Introduction

Hepatocellular carcinoma (HCC) is one of the most common cancers worldwide, with an increasing incidence, rapid progression and frequent tumor recurrence and metastasis [1,2,3]. Colorectal cancer is the third most common cancer worldwide, and approximately 20%–30% of these patients will develop liver metastasis during their lifetime [4,5]. For both hepatocellular carcinoma and colorectal liver metastases (CRLM), hepatic resection (hepatectomy) remains the mainstay intervention with curative intent, followed by modern techniques, such as orthotopic liver transplantation or transarterial chemoembolization, which emerge nowadays. According to Furukawa et al., hepatic resection can provide a prolonged survival for patients with CRLM, with a 5-year survival rate up to 30%–60%. However, even in patients with curative hepatic resections, there is still a high recurrence rate postoperatively (up to 70% in a 5-year follow-up) and a high morbidity rate (approximately 40%–50%) [1,2,6,7]. Many risk factors, such as sarcopenia, are closely related to high incidence of postoperative complications and poor long-term outcomes for patients with cancer [3]. Therefore, early preoperative recognition of perioperative risk factors is crucial in avoiding adverse consequences after hepatectomy and improving overall survival (OS) and disease-free survival (DFS) for these patients.

Sarcopenia is a term first introduced by Rosenberg in 1989 to describe the involuntary age-related loss of skeletal muscle mass [4,8,9]. It was initially noticed in elderly people and had a negative impact on health. In 2010, The European Working Group on Sarcopenia in Older People (EWGSOP) published a clinical definition of sarcopenia, which described it as a syndrome characterized by progressive and generalized loss of skeletal muscle mass (quality and quantity), strength and function [4,6,7,8,10,11].

Sarcopenia is strongly associated with nutritional health/malnutrition and is of great prognostic significance in patients with cancer [1,6]. Malnutrition is very common in these patients due to the combination of malignant disease progress and anticancer treatment, and in severe cases, it can lead to cachexia, a syndrome characterized by the loss of skeletal muscle mass. It is highly associated with tumor aggressiveness, longer hospitalization, and it is identified as a poor prognostic factor with reduced overall, disease-free and recurrence-free survival rate for patients with cancer undergoing surgery [1,4,10,12,13,14]. Sarcopenia also carries a risk of increased short-term and long-term adverse outcomes, such as major postoperative complications, physical disability, poor health-related quality of life, postoperative morbidity and death [2,10,15,16]. According to Hou et al.’s retrospective study published in 2021 comparing sarcopenic and non-sarcopenic patients with HCC and cholangiocarcinoma undergoing liver resection, sarcopenia has been proven to be an independent prognostic indicator for overall survival and disease-free survival for these patients after surgery. This finding agrees with other previous studies indicating that sarcopenia is related to adverse postoperative results and poor prognosis for cancer patients, as described in a study by Bernardi et al. in 2020, in which sarcopenic patients undergoing hepatectomies presented a higher 90-day morbidity rate compared to non-sarcopenic patients, as well as a higher complication incidence.

Body composition profiling plays a valuable role in preoperative risk assessment and in predicting the short-term and long-term outcomes of patients undergoing oncologic liver surgery [17,18]. Based on parameters obtained from diagnostic preoperative imaging with computed tomography (CT) or magnetic resonance imaging (MRI), such as the measurement of psoas area, muscle density at the third lumbar vertebra (L3) and intramuscular adipose tissue in Hounsfield units, it is possible to accurately quantify intra-abdominal fat and muscle mass in order to reveal sarcopenia and predict postoperative survival rate after surgical resection for colorectal cancer, hepatocellular carcinoma and colorectal liver metastasis [5,10]. Any abnormalities in these parameters are associated with poor postoperative outcomes and prognosis.

This systematic review aims to summarize the current evidence available from the literature on the effects of sarcopenia on overall survival and postoperative complications regarding liver resections in patients suffering from primary liver or metastatic colorectal cancer.

## 2. Materials and Methods

### 2.1. Study Selection

**Figure 1:**
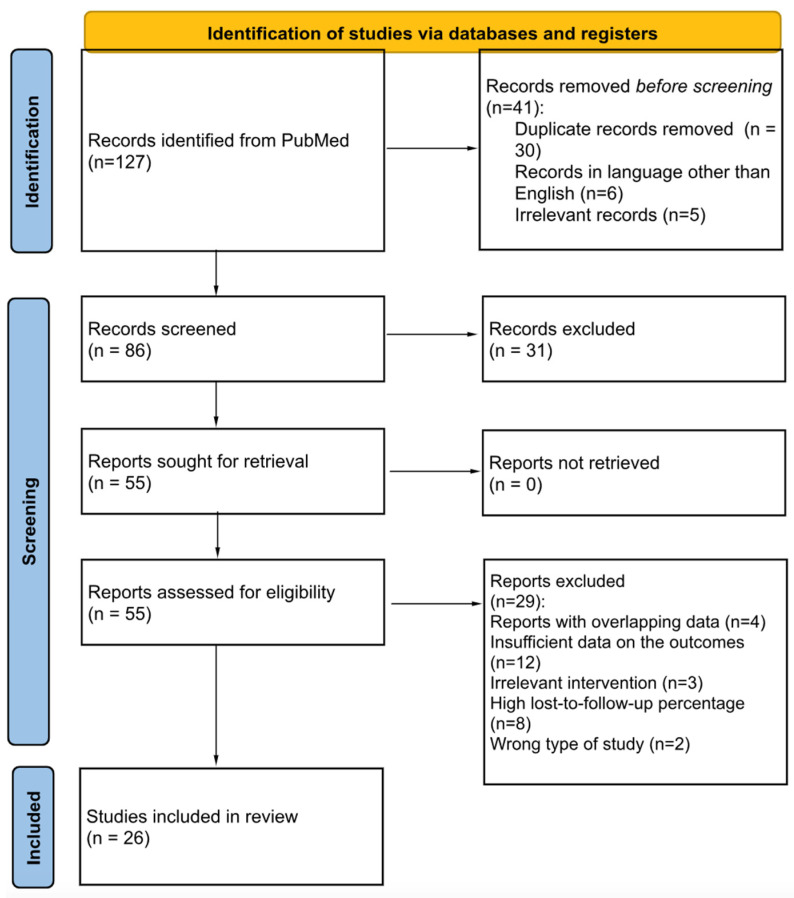
PRISMA flow diagram.

A thorough literature search was conducted on PubMed for articles including patients with sarcopenia undergoing liver resection for primary or metastatic liver cancer. The terms “sarcopenia”, “liver resection”, “hepatectomy”, “metastasis”, “metastases”, “hepatocellular carcinoma”, “hcc”, “laparoscopic liver resection” and “complications” were used in various combinations. The search was conducted manually by two independent reviewers and yielded 209 results. Any conflict during the selection process was resolved through discussion. All articles were scrutinized against predetermined inclusion and exclusion criteria, and after excluding duplicates and irrelevant studies, 86 were eligible for further assessment. After full-text screening, 60 studies were excluded and 26 were finally included in our systematic review. The study selection algorithm is shown on the Preferred Reporting Items of Systematic Reviews and Meta-Analyses (PRISMA) flow chart (Figure 1) [19]. The systematic review protocol was registered in the International Prospective Register of Systematic Reviews (PROSPERO ID CRD42023426589).

### 2.2. Inclusion and Exclusion Criteria

The following inclusion criteria were applied: cohort studies with adult patients published over the last decade in the English language; studies including patients undergoing open or laparoscopic hepatic resections for HCC or CRLM; studies including patients with sarcopenia, as defined above, prior to any intervention related to the hepatic disease; studies having overall survival or complications after hepatectomy as primary outcomes.

Case reports, case series, commentaries and letters to the editor were excluded from this review.

### 2.3. Definition

The International Working Group on Sarcopenia (IWGS) has published a consensus in which sarcopenia is defined as the presence of low skeletal muscle mass and low muscle function [20]. However, other associations, such as the European Working Group on Sarcopenia in Older People (EWGSOP) and the European Society for Clinical Nutrition and Metabolism—Special Interest Groups (ESPEN—SIG), have proposed their own sarcopenia definitions, defining it, respectively, as (i) the presence of low skeletal muscle mass and either low muscle strength (assessed by handgrip) or low muscle performance (assessed by measuring the walking speed) and (ii) the presence of low skeletal muscle mass and low muscle strength (assessed by handgrip) [21,22]. In our review, the skeletal muscle mass was assessed with preoperative CT scans at the L3 level, except for one study that measured the cross-sectional muscle area at the L4 level [5]. The total muscle area was then normalized for height, generating the skeletal muscle index (SMI). Sarcopenia was finally defined based on the SMI cut-off values that each study set according to international consensuses or statistical analyses, which differed among the studies and between men and women.

### 2.4. Data Extraction

The following data were extracted in a preformed datasheet: author, year, institution and study period, type of operation, patient population, age, sex, BMI, primary disease, staging, administration of neoadjuvant chemotherapy (NAC), follow-up, overall survival, morbidity, mortality and postoperative complications. 

### 2.5. Risk of Bias and Quality Assessment

The risk of bias and the quality of each individual study were assessed using the Cochrane Tool to Assess Risk of Bias in Cohort Studies and the Newcastle–Ottawa Quality Assessment Scale (NOS), respectively [23]. The Cochrane Tool consists of 7 questions, and according to the answers, a cohort study can be categorized as having low or high risk of bias. The NOS consists of 8 items regarding the selection of subjects, the comparability and the outcomes of each individual cohort study (Table 1).

### 2.6. Meta-Analysis

The studies that were included in the analysis were reviewed, and the data were tabulated. Due to differences in the methodology used in each study, not all of the examined data were available in every study. The data were then organized into groups and inputted into the SPSS platform, which provided the results subsequently analyzed in detail below.

Due to the large number of studies in the meta-analysis, it was necessary to sub-categorize them for better statistical management. Therefore, the studies were grouped based on the parameters analyzed to yield more significant statistical results.

**Figure 2:**
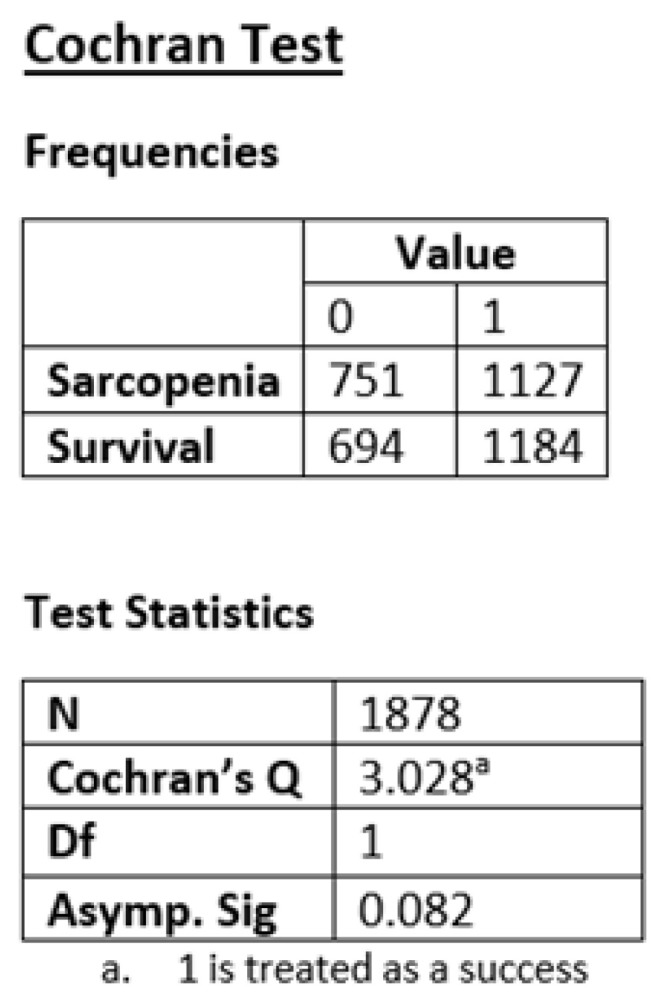
Cochran’s Q Test.

The null hypothesis for Cochran’s Q is that the percentage of “hits” is equal for all groups. The alternative hypothesis, on the other hand, suggests that the ratio varies at least among one group. If the calculated critical value Q is greater than a critical χ^2^ value, then the null hypothesis is rejected. If the variation is only due to within-study error, then its expected value would be the degrees of freedom for this meta-analysis, where df = 25. Therefore, it follows that Q < df. By calculating I^2^, it shows that I^2^ = 0, indicating that the heterogeneity of the sample is not statistically significant for the meta-analysis (Figure 2).

The research studies were divided into categories based on their focus on the complication rate in sarcopenic patients. By analyzing these groups, the odds ratio (OR) of sarcopenic patients who developed complications in the various studies was determined, and a forest plot was created for the studies included.

## 3. Results

In this study, a total of 6103 patients underwent hepatic resection of one or more segments or metastasectomy. The study aimed to investigate the effect of sarcopenia on the survival, mortality and complication rate of the patients. 

The study analyzed patients based on their sex, age and whether they had sarcopenia. The data were also categorized based on the stage of the disease and the initial location of the tumor, as well as the presence of preoperative chemotherapy. The patients’ demographics and characteristics are shown in Table 2. The patients were followed up for varying periods, and their postoperative complications, mortality and survival were studied.

After collecting the data, statistical analysis was conducted, and several results were extracted. Out of the 6103 patients included in the study, 2232 were diagnosed with sarcopenia, while 3841 were not. Among the total number of patients, 2167 were male, and 1126 were female, resulting in a male-to-female ratio of 2:1. The average age of all patients was 64.3 years. The average BMI for patients with sarcopenia was 22.9, whereas for non-sarcopenic patients, it was 25.8. On average, the SMI of sarcopenic individuals was 38.9, while for non-sarcopenic patients, it was 49.6.

**Figure 3:**
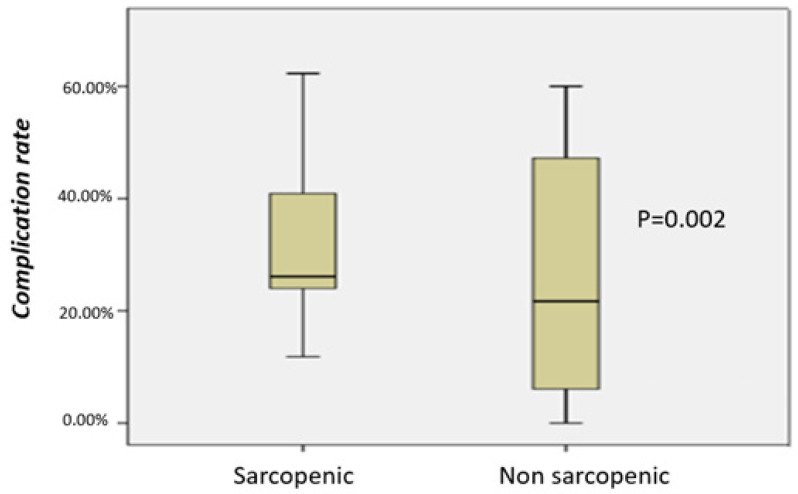
Complication rate of sarcopenic vs. non-sarcopenic patients.

**Figure 4:**
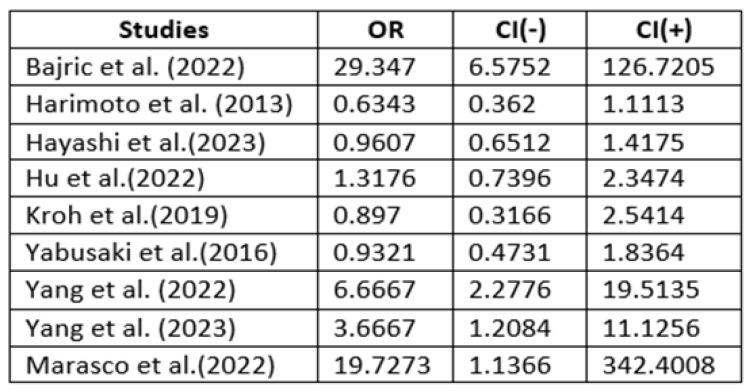
Odds ratio of complication rate in sarcopenic patients. OR, odds ratio [2,3,10,14,16,24,25,30,31].

**Figure 5:**
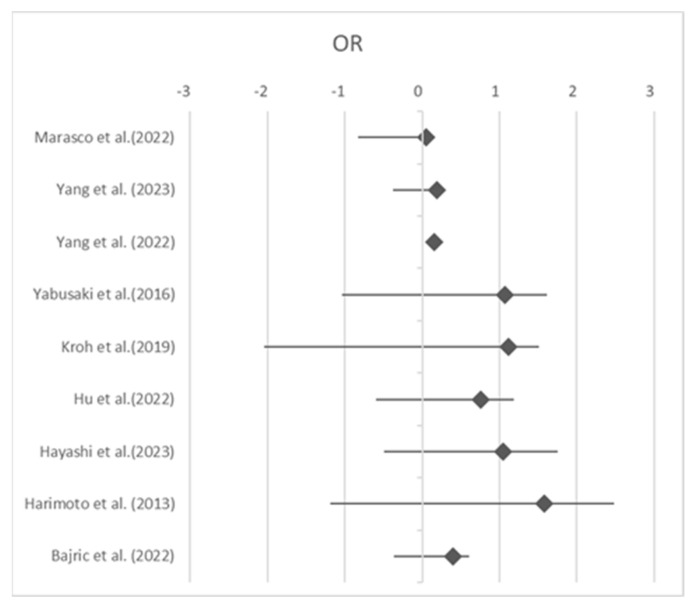
Forest plot. OR, odds ratio [2,3,10,14,16,24,25,30,31].

According to the data, the average rate of complications in patients with sarcopenia is 35.4%, while in non-sarcopenic patients, it is 23.1% (35.4% vs. 23.1%; *p* = 0.002). The diagram in Figure 3 shows that the complication rate in sarcopenic patients is significantly higher than in non-sarcopenic patients. Furthermore, Figure 4 and Figure 5 show the OR of the complication rates in the nine studies that compared the complications between sarcopenic and non-sarcopenic patients. However, it was not possible to classify the complications further based on Clavien–Dindo classification, as it was only used in very few studies. The morbidity of sarcopenic patients in comparison to non-sarcopenic patients and the specific complications and their incidence reported in each of the included studies are shown in Table 3 and Table 4.

No further statistical analysis could be performed on complications experienced by patients, as they were not further analyzed in most studies. The most commonly reported complications include surgical wound infection and delayed gastric emptying. In the studies that do provide information regarding complications, they are typically classified using the Clavien–Dindo system, with the majority falling into grade I or II.

The average 5-year OS rate of the patients included in our study was 64.7%. The patients who were diagnosed with sarcopenia had an average OS rate of 43.8%, which ranged from 13.4% to 91.1%. On the other hand, the non-sarcopenic patients had an average OS rate of 63.6%, ranging from 9.7% to 99.1%. Sarcopenic patients had a statistically significantly lower 5-year OS than non-sarcopenic patients, with a *p*-value < 0.01. No statistical correlation was found between mortality incidence in sarcopenic and non-sarcopenic patients (*p*-value > 0.1) (Table 5).

A sub-categorization was created to include studies that included the rate of complications experienced by patients after hepatectomy. Additionally, data evaluation on the initial location of the tumor and the occurrence of sarcopenia led to the conclusion that patients with hepatocellular carcinoma or liver metastasis do not exhibit a statistically significant difference in the occurrence of sarcopenia (*p*-value > 0.01). However, a significant difference is observed in patients who underwent hepatectomy due to a tumor in the right or left colon. Patients who underwent right colectomy are more likely to experience sarcopenia as compared to those who underwent left colectomy or low anterior resection. This difference can be attributed to the removal of the cecum and terminal ileum during the right colectomy. 

This was followed by the survival study of sarcopenic and non-sarcopenic patients after hepatectomy. We should take into account that the studies were conducted in different time periods, and the follow-up period of these patients was different in every study.

**Figure 6:**
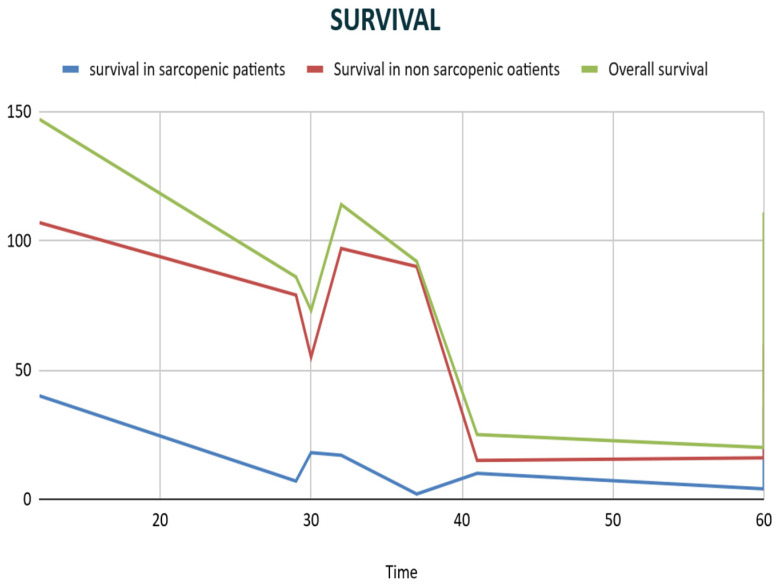
Kaplan–Meier curve.

As is evident from the diagram in Figure 6, a lower survival rate appears in sarcopenic patients compared to non-sarcopenic patients. At 30 months of follow-up, there is an increase in the line, which is not actually the case, as there were two studies that had the same follow-up months with different results in terms of survival rates. The Log-rank test was 1.0146, with a *p*-value 0.3131.

**Figure 7:**
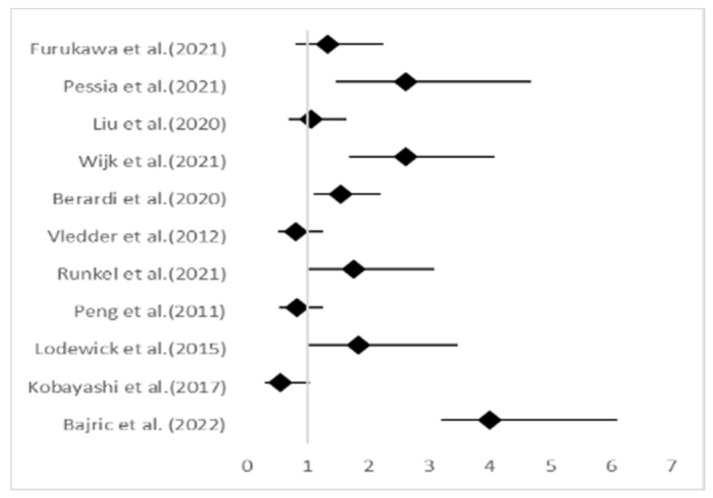
Forest plot for right and left colon [4,5,7,8,9,24,26,27,28,35,37].

The subgroup of patients who underwent hepatectomy for metastatic colorectal cancer was then created. It was studied in terms of the odds ratio (Table 6), and then, a forest plot (Figure 7) was created, wherein those studies that included this subgroup were distinguished.

## 4. Discussion

In our systematic review, we assessed the effects of preoperative sarcopenia on the survival and postoperative complications of patients undergoing hepatectomy for HCC or CRLM. The meta-analysis showed that OS was statistically significantly shorter in sarcopenic patients compared to non-sarcopenic patients and that patients with preoperative sarcopenia had a statistically significantly higher complication rate than non-sarcopenic patients. Regarding postoperative mortality, no difference was reported between the two groups of patients. Interestingly, patients who had undergone right colectomy were more likely to develop sarcopenia compared to those who had undergone left colectomy. During right colectomy, the cecum and terminal ileum are resected, which are important parts of the large intestine for absorbing water, electrolytes and nutrients. Therefore, special attention should be paid to the postoperative nutrition of these patients, which is addressed later in this section. Short OS and increased morbidity of patients undergoing hepatectomy for liver metastases were also reported by O’Connel et al. in their meta-analysis. At the same time, they noted that obesity was not associated with worse oncological outcomes [39]. Poor prognosis of patients with sarcopenia treated for HCC was also reported in a recent meta-analysis by Kong et al. [40]. Furthermore, Thormann et al. concluded that low skeletal muscle mass led to significantly more postoperative complications after surgery for hepatic metastases but not after surgery for HCC or cholangiocarcinoma [41]. Conversely, Erikson et al. did not find a correlation between muscle loss and worse OS in their study of patients treated for colorectal liver metastases [17]. The different conclusions may be explained when considering that other factors, such as muscle strength or physical performance of the patients, could also affect the perioperative risk [42]. However, sarcopenia has been associated with short OS and high rates of complications in various studies in the literature, including patients with other hepatopancreatobiliary malignancies, gastric, colorectal or small-cell lung cancer [43,44,45,46,47].

In order to diagnose sarcopenia, the muscle mass of the patient needs to be calculated first. A CT scan is used to measure muscle density at the level of the third lumbar vertebra, which is then normalized for the patient’s height. The resulting value is known as the SMI. A patient with an SMI below a certain cut-off value is characterized as sarcopenic. Various means of determining an SMI cut-off value have been described in the literature. The most common method is using data from healthy individuals to calculate the mean SMI. Sarcopenia is defined as more than two standard deviations below the mean [48,49]. Another means commonly used both in the literature and in the studies included in our review is optimal stratification of the data, which results in the ideal cut-off value for both men and women [28,50]. Even though the methods for cut-off calculation are standard, the cut-off value itself varies greatly among the different studies. For instance, some SMI cut-offs used for patients with respiratory or gastrointestinal tract tumors were 38.5, 30.88 or 41.1 for women and 52.4, 40.33 or 43.75 for men [28,48,49,50]. Efforts should be made to introduce a standard and universal sarcopenia definition, so that the effects of sarcopenia on patients with cancer can be more systematically assessed.

Another confounding factor is that the administration of NAC in patients undergoing curative intent surgery for hepatic malignancies is not systematically addressed in the literature. In our review, only 10 out of 26 studies reported the administration of neoadjuvant chemotherapy, as shown in Table 2, while in 2 out of 10 studies [29,34], none of the patients received systematic therapy. Even though the administration of preoperative chemotherapy is similar among sarcopenic and non-sarcopenic patients, there are not enough data regarding the tolerance or completion of it [18]. Furthermore, loss of skeletal muscle has been reported after NAC, which may further worsen the performance status of sarcopenic individuals and lead to poor outcomes. This was shown in a study by Miyamoto et al. after NAC administration for unresectable colorectal cancer [51]. However, Eriksson et al. did not report worse OS after NAC in patients with resectable colorectal liver metastases who had lost over 5% of skeletal muscle during therapy [17]. It is evident that chemotherapy may indeed affect the patient’s preoperative status, but unfortunately, insufficient data are available in the literature. Future studies should focus on reporting the effects of chemotherapy on preoperative nutritional status, so that the timing of the operation and the perioperative support can be optimized.

Sarcopenia is frequent in patients with cancer and especially in patients with liver cancer. Its impact on survival dictates the necessity for adequate prevention, which is challenging due to its multifactorial nature [52]. Body composition, such as myosteatosis, central or sarcopenic obesity and visceral fat amount seem to gain popularity as predictors of survival too, which implies the critical role of nutritional status [7,35]. Two major strategies have been described to improve body composition. The first is nutritional therapy, which includes preoperatively adding branched-chain amino acids, leucine, lipids, dextrose and L-carnitine to the patient’s diet. Fan et al. divided their patients undergoing hepatectomy for HCC into two groups: one who received nutritional support and one control group. They showed that the worsening of liver function, sepsis-related complications and overall postoperative morbidity were lower in the nutrition compared to the control group (34% vs. 55%) [53]. The second is physical activity, which could inhibit the progress of sarcopenia by recruiting more myofibers, although reversing it remains unclear. Another promising method to reverse sarcopenia is the use of selective androgen receptor modulators (SARMs). Due to their anabolic effects, SARMs increase bone and muscle mass and inhibit protein degradation, decreasing sarcopenia’s progression rate [54]. To conclude, multidisciplinary support should be considered in select cases of sarcopenic patients who undergo surgical and systematic therapy in order to improve or even reverse sarcopenia and optimize the outcomes.

One limitation of our systematic review is that the studies included were retrospective cohort studies, thereby prone to recall or selection bias. Another limitation is that there are no standard SMI cut-offs for diagnosing sarcopenia. Even when the same statistical method was applied to determine the cut-offs, different thresholds occurred in different populations. Furthermore, patients did not undergo the same type of hepatectomy, which may have affected survival, as larger operations tend to have more complications. Of note, operations were performed in different institutes by different teams with variable experience. In our study, we included patients with both primary and metastatic liver disease. A more advanced disease is by nature a poor prognostic factor, and patients with metastatic disease usually present with worse performance status, which in turn may affect the postoperative outcomes independently. Of note, none of the studies reported the administration of preoperative nutritional support. Finally, some patients received neoadjuvant chemotherapy, but the timing from last therapy to surgery was not available. Also, the follow-up varied greatly among the studies and was unavailable in many of them. In future, studies should focus on standardizing sarcopenia assessment and determining universal cut-off values based on data from large populations, as well as on incorporating methods for improvement of preoperative patient status in order to optimize patient care.

## 5. Conclusions

In conclusion, sarcopenia could lead to poor OS and high complication rates in patients undergoing hepatectomy for HCC or CRLM. We showed that sarcopenic patients had a significantly decreased OS and significantly more postoperative complications compared to non-sarcopenic patients. However, by integrating perioperative nutritional support and physical activity, the effects of sarcopenia could be reversed, with potentially improved postoperative outcomes. Future research should focus on conducting large prospective studies in high-volume centers with standardized protocols in an attempt to achieve optimal results for select patients with liver malignancy undergoing curative intent surgery.

## Figures and Tables

**Table 1:** Newcastle–Ottawa Scale scores for the included studies. * is a star awarded for each item.

Study	Selection				Comparability	Outcomes			Total
	Representativeness of the Exposed Cohort	Selection of the Non-Exposed Cohort	Ascertainment of Exposure	Outcome of Interest Not Present at the Start of the Study		Assessment of Outcome	Length of Follow-Up	Adequacy of Follow-Up	[24]
Bajric et al. [24]	*	*	*	*	*	*	*	*	8/8
Harimoto et al. [2]	*	*	*	*	*	*		*	7/8
Harimoto et al. [11]	*		*	*		*			4/8
Hayashi et al. [3]	*	*	*	*	*	*	*	*	8/8
Hu et al. [16]	*	*	*	*	*	*	*		7/8
Kobayashi et al. [4]	*	*	*	*	*	*			6/8
Kroh et al. [25]	*	*	*	*	*	*	*	*	8/8
Lodewick et al. [26]	*	*	*	*	*	*			6/8
Peng et al. [27]	*	*	*	*		*			5/8
Runkel et al. [9]	*		*	*		*			4/8
VanVledder et al. [28]	*	*	*	*	*	*	*	*	8/8
Wu et al. [29]	*	*	*	*	*	*			6/8
Yabusaki et al. [30]	*	*	*	*	*	*	*	*	8/8
Yang et al. [14]	*	*	*	*	*	*			6/8
Yang et al. [31]	*	*	*	*	*	*			6/8
Berardi et al. [7]	*	*	*	*	*	*			6/8
Kim et al. [32]	*	*	*	*		*			5/8
Marasco et al. [10]	*	*	*	*	*	*	*	*	8/8
Hou et al. [33]	*	*	*	*		*	*	*	7/8
Zhou et al. [34]	*	*	*	*	*	*			6/8
Wijk et al. [35]	*	*	*	*		*			5/8
Liu et al. (2020) [5]	*	*	*	*		*	*	*	7/8
Xiong et al. [36]	*	*	*	*		*	*	*	7/8
Pessia et al. [37]	*	*	*	*	*	*	*	*	8/8
Xu et al. [38]	*	*	*	*	*	*	*	*	8/8
Furukawa et al. [8]	*	*	*	*	*	*	*	*	8/8

**Table 2:** Patient demographics and characteristics.

Author	Population	Groups	Sex	Age (mean)	BMI (mg/m^2^)	SMI	Primary Tumor Location	Tumor Stage	Neoadjuvant Chemotherapy
Bajric et al. [24]	315	Sarcopenic, n = 78 (24.7%); non- sarcopenic, n = 237 (75.3%)	135 M, 220 F	68 (60–74)	25.5 (23.3–28.7)	NA	Colorectal cancer	IV (100%)	NA
Harimoto et al. [2]	186	Sarcopenic, n = 75 (40.3%); non-sarcopenic, n = 111 (59.7%)	145 M, 41 F	66 (55–77)	Sarcopenic, 20.5 (18.1); non-sarcopenic, 24 (21.2–26.8); *p* < 0.001	Sarcopenic, 37.8; non-sarcopenic, 49.7; *p* < 0.001	Liver	Sarcopenic, I n = 11 (14.7%), II n = 38 (50.7%), III n = 20 (26.6%), IV n = 6 (8%); non-sarcopenic, I n = 18 (16.2%), II n = 57 (51.3%), III n = 29 (26.2%), IV n = 7 (6.3%)	NA
Harimoto et al. [11]	146	Sarcopenic, n = 146	106 M, 40 F	68 (28–89)	22.7 (14.8–31.4)	36.8	Liver	NA	NA
Hayashiet al. [3]	303	Sarcopenic, n = 106 (34.9%); non-sarcopenic n = 197 (65.1%)	Sarcopenic, 96 M/10 F; non-sarcopenic, 125 M/72 F	Sarcopenic, 72 (38–89); non-sarcopenic, 70 (36–85)	Sarcopenic, n = 21.9 (13.4–32.5); non-sarcopenic, 23.8 (16.5–45.2); *p* < 0.001	NA	Liver	Sarcopenic, I n = 20 (18.9%), II n = 41 (38.7%), III n = 34 (32.1%), IV n = 11 (10.3%); non-sarcopenic, I n = 49 (24.9%), II n = 84 (42.6%), III n = 50 (25.4%), IV n = 14 (7.1%)	NA
Hu et al. [16]	153	Sarcopenic, n = 45 (29.4%); non-sarcopenic, n = 108 (70.6%)	133 M, 45 F	60 (51–66)	<25, n = 109; >25, n = 44	Sarcopenic: 41.84; non-sarcopenic: 49.20 (*p* < 0.001)	Liver	I n = 95 (62.1%), II–IV n = 58 (37.9%)	NA
Kobayashi et al. [4]	124	Sarcopenic, n = 24 (19.3%); non-sarcopenic, n = 100 (80.7%)	78 M, 46 F	65 (59–70)	22.7 (20.3–24.7)	NA	Colon, n = 69; Rectum, n = 55	I/II, n = 28 (24%); III/IV, n = 91 (76%)	52%
Kroh et al. [25]	70	Sarcopenic, n = 33 (47.1%); non-sarcopenic, n = 37 (52.9%)	49 M, 21 F	67 (54–80)	26.64 (22.02–31.26)	47.98	Liver	T1, n = 22 (31.5%) T2, n = 25 (35.7%) T3, n = 23 (32.8%) *	NA
Lodewick et al. [26]	80	Sarcopenic, n = 31 (38.7%); non-sarcopenic, n = 49 (61.3%)	51 M, 29 F	66 (28–82)	24.9 (18.7–46.4)	NA	Colorectal/HBC	NA	NA
Peng et al. [27]	259	Sarcopenic, n = 41 (15.8%); non-sarcopenic, n = 218 (84.2%)	155 M, 104 F	58 (46–70)	<30, n = 191; ≥30, n = 68	NA	Colon, n = 191; Rectum, n = 68	T1/T2, n = 41 (15.8%) T3/T4, n = 218 (84.2%) *	NA
Runkel et al. [9]	94	Sarcopenic, n = 94	58 M, 36 F	61 (34–83)	26 (13.8–45.6)	NA	Colorectal cancer	IV (100%)	62.8%
VanVledder et al. [28]	196	Sarcopenic, n = 38 (19.4%); non-sarcopenic, n = 158 (80.6%)	120 M, 76 F	64.5 (31–86)	Sarcopenic, 23.7 (20.7–26.7); non-sarcopenic, 26.7 (23.2–30.2)	NA	Colon, n = 116; Rectum, n = 80	T2 n = 25 (13.2%), T3 n = 148 (78.3%), T4 n = 16 (8.5%) *	Sarcopenic, 47%; non-sarcopenic, 46.2%
Wu et al. [29]	1172	Sarcopenic, n = 421 (35.9%); non-sarcopenic, n = 751 (65.1%)		Sarcopenic: <65, n = 329; ≥65, n = 92; non-sarcopenic: <65, n = 613; ≥65, n = 138	Sarcopenic, 25.47 (21.77–26.32); non-sarcopenic, 22.94 (20.76–25.63) *p* < 0.001	Sarcopenic, 37.84; non-sarcopenic, 46.68 *p* < 0.001	HCC	Sarcopenic, I n = 191 (45.3%), II n = 100 (23.7%), III n = 121 (28.8%), IV n = 9 (2.2%); non-sarcopenic, I n = 342 (45.6%), II n = 180 (23.9%), III n = 217 (28.9%), IV n = 12 (1.6%)	None
Yabusaki et al. [30]	195	Sarcopenic, n = 89 (45.6%); non-sarcopenic, n = 106 (54.4%)	157 M, 38 F	66 (22–80)	23.2 (14.3–37.3)	NA	HCC	I, n = 20 (10.3%) II, n = 112 (57.4%) III, n = 42 (21.5%) IVA, n = 19 (9.7%) IVB, n = 2 (1.1%)	NA
Yang et al. [14]	155	Sarcopenic, n = 89 (57.4%); non-sarcopenic, n = 66 (42.6%)	135 M, 20 F	60 (51–66)	23.37 (23.14–23.6)	47.05	HCC	I–II, n = 138 (89.1%) III–IV, n = 17 (10.9%)	NA
Yang et al. [31]	171	Sarcopenic, n = 86 (50.2%); non-sarcopenic, n = 85 (49.8%)	99 M, 72 F	59 (50–67)	22.86 (20.94–25.08)	42.22	HCC, n = 47 CLM, n = 44 Other HBC **, n = 80	NA	NA
Berardi et al. [7]	234	Sarcopenic, n = 143 (61.2%); non-sarcopenic, n = 91 (38.8%)	158 M, 76 F	66 (58–74)	27.12 (23,28–29,55)	46.22	HCC, n = 101; Colorectal cancer, n = 96	NA	Sarcopenic, 36.4%; non-sarcopenic, 39.5%
Kim et al. [32]	159	Sarcopenic, n = 74 (46.5%); non-sarcopenic, n = 85 (53.5%)	133 M, 26 F	59 (49–69)	24.8 (21.1–28.42)	51.08	HCC	I–II, n = 65 (40.9%); III–IV, n = 94 (59.1%)	NA
Marasco et al. [10]	159	Sarcopenic, n = 82 (51.6%); non-sarcopenic, n = 77 (48.4%)	128 M, 31 F	68 (58–75)	Sarcopenic, 25.6 (23.8–27.8); non-sarcopenic, 27.5 (25.6–29.4)	F: 39.2; M: 48.9	HCC	NA	NA
Hou et al. [33]	153	Sarcopenic, n = 77 (50.3%); non-sarcopenic, n = 76 (49.7%)	128 M, 25 F	>55, n = 68; ≤55, n = 85	Sarcopenic, 21.64 (19.73–23.78); non-sarcopenic, 24.27 (21.93–25.62)	NA	Combined HCC–CC	HCC: Stage I n = 21 (13.7%), stage II n = 18 (11.8%), stage III n = 92 (60.1%), stage IV n = 22 (14.4%); CC: Stage I n = 22 (14.4%), stage II n = 23 (15%), stage III n = 108 (70.6%)	NA
Zhou et al. [34]	67	Sarcopenic, n = 33 (49.3%); non-sarcopenic, n = 34 (50.7%)	22 M, 45 F	61 (47–81)	22.2 (24.4–28.7)	41.2	IHCC	I–II, n = 44 (65.7%); III–IV, n = 23 (34.3%)	None
Wijk et al. [35]	128	Sarcopenic, n = 83 (64.8%); non-sarcopenic, n = 45 (35.2%)	89 M, 39 F	65.5 (57–74)	25.6 (22.5–28.7)	NA	Colorectal cancer	NA	NA
Liu et al. [5]	182	Sarcopenic, n = 48 (26.4%); non-sarcopenic, n = 134 (73.6%)	106 M, 76 F	59.5 (28–85)	24.3 (20.7–27.9)	NA	Colorectal cancer	T1 n = 3 (1.6%), T2 n = 22 (12.1%), T3 n = 73 (40.1%), T4 n = 84 (46.2%) *	Sarcopenic, 21%; non-sarcopenic, 19%
Xiong et al. [36]	114	Sarcopenic, n = 58 (50.8%); non-sarcopenic, n = 56 (49.2%)	91 M, 23 F	62.5 (57–70)	<18.5, n = 20; ≥18.5, n = 94	Sarcopenic, 34.2; non-sarcopenic, 42.7	Gastric cancer	NA	Sarcopenic, 53.5%; non-sarcopenic, 58.9%
Pessia et al. [37]	74	Sarcopenic, n = 48 (64.8%); non-sarcopenic, n = 26 (35.2%)	NA	NA	Sarcopenic, 24.2; non-sarcopenic, 27.6	Sarcopenic, 39.3; non-sarcopenic, 52.7	Colorectal cancer	NA	100%
Xu et al. [38]	1420	Sarcopenic, n = 458 (32.2%); non-sarcopenic, n = 962 (67.8%)	NA	NA	Sarcopenic, 24.2; non-sarcopenic, 27.6	Sarcopenic, 39.3; non-sarcopenic, 52.7	HCC	NA	NA
Furukawa et al. [8]	63	Sarcopenic, n = 33 (52.3%); non-sarcopenic, n = 30 (47.7%)	31 M, 37 F	67.5 (28–90)	NA	NA	Colorectal cancer	NA	Sarcopenic, 37%; non-sarcopenic, 34%

M, male; F, female; NA, not applicable; HBC, hepatobiliary carcinoma; HCC, hepatocellular carcinoma; CLM, colorectal liver metastases; CC, cholangiocarcinoma; IHCC, intrahepatic cholangiocarcinoma. * The AJCC TNM system was used for HCC, HBC, CC or IHCC staging. When information on lymph nodes (N) or metastasis (M) was not available, only the tumor extent (T) was used to present the data. ** Cholangiocarcinoma, n = 67, Gallbladder cancer, n = 7, Mixed liver cancer, n = 6.

**Table 3:** Morbidity of sarcopenic vs. non-sarcopenic patients undergoing hepatectomy for liver cancer.

Study		Population	Complication Rate	Follow-Up (Months)
Bajric et al. [24]		315		30
	Sarcopenic	78 (24.7%)	24.9%	
	Non-sarcopenic	237 (75.3%)	9.7%	
			*p* = 0.01	
Harimoto et al. [2]		186		NA
	Sarcopenic	75 (40.3%)	32%	
	Non-sarcopenic	111 (59.7%)	50.5%	
			*p* = 0.613	
Harimoto et al. [11]		146		NA
	Sarcopenic	146	8.2%	
	Non-sarcopenic	0	-	
			-	
Hayashi et al. [3]		303		60
	Sarcopenic	106 (34.9%)	58%	
	Non-sarcopenic	197 (65.1%)	60%	
			*p* = 0.812	
Hu et al. [16]		153		12
	Sarcopenic	45 (29.4%)	62.3%	
	Non-sarcopenic	108 (70.6%)	47.2%	
			*p* = 0.162	
Kroh et al. [25]		70		60
	Sarcopenic	33 (47.1%)	24%	
	Non-sarcopenic	37 (52.9%)	27%	
			*p* = 1	
Peng et al. [27]		249	23%	NA
	Sarcopenic	41 (15.8%)	-	
	Non-sarcopenic	218 (84.2%)	-	
			-	
Runkel et al. [9]		94		NA
	Sarcopenic	94	62.8%	
	Non-sarcopenic	0	-	
			-	
Wu et al. [29]		1172		NA
	Sarcopenic	421 (35.9%)	*Significantly higher*	
	Non-sarcopenic	751 (65.1%)	-	
			*p* < 0.001	
Yabusaki et al. [30]		195		37
	Sarcopenic	89 (45.6%)	20.2%	
	Non-sarcopenic	106 (54.4%)	21.7%	
			*p* = 0.8	
Yang et al. [14]		155		NA
	Sarcopenic	89 (57%)	40.9%	
	Non-sarcopenic	66 (43%)	6.06%	
			*p* < 0.001	
Yang et al. [31]		171		NA
	Sarcopenic	86 (50.2%)	26.1%	
	Non-sarcopenic	85 (49.8%	4.5%	
			*p* = 0.032	
Berardi et al. [7]		234	30.3%	3
	Sarcopenic	91 (38.8%)	-	
	Non-sarcopenic	143 (61.2%)	-	
				
Marasco et al. [10]		159		30
	Sarcopenic	82 (51.6%)	11.8%	
	Non-sarcopenic	77 (48.4%)	0	
			*p* = 0.032	
Wijk et al. [35]		128	40.6%	NA
	Sarcopenic	83 (64.8%)	-	
	Non-sarcopenic	45 (35.2%)	-	
			-	
Kim et al. [32]		159	41.5%	1
	Sarcopenic		-	
	Non-sarcopenic		-	
			-	
Liu et al. [5]		182	33%	32.5
	Sarcopenic		38.3%	
	Non-sarcopenic		27.7%	
			-	
Xiong et al. [36]		114	29.3%	60
	Sarcopenic		43.7%	
	Non-sarcopenic		33.6%	
			-	

**Table 4:** Complications reported after hepatectomy.

Complications after Hepatectomy			
Complication	Number of Cases	Complication	Number of Cases
Bile leakage	83	Liver failure	109
Ascites	8	Pleural effusion	80
Pneumonia	4	Surgical site infection	50
Intra-abdominal abscess	62	Postoperative bleeding	10
Brain infarction	1	Hepatic encephalopathy	1
Obstruction of blood dialysis shunt	1	Reintubation	1
Biloma	2	Sepsis	1
Portal vein thrombosis	1	Cardiopulmonary	3
Gastrointestinal	2	Hematological	3
Bacteremia	2	Miscellaneous infections	6

**Table 5:** 5-year overall survival and mortality of sarcopenic vs. non-sarcopenic patients undergoing hepatectomy for liver cancer.

Study		Population	5-Year Overall Survival	Mortality (30 Days)	Follow-Up (Months)
Bajric et al. [24]		315			30
	Sarcopenic	78 (25%)	20.3%	38.2%	
	Non-sarcopenic	237 (75%)	23.1%	34.3%	
			*p* = 0.01	*p* > 0.05	
Harimoto et al. [2]		186			NA
	Sarcopenic	75 (40.3%)	71%	-	
	Non-sarcopenic	111 (59.7%)	83.7%	-	
			*p* = 0.001		
Hayashi et al. [3]		303			60
	Sarcopenic	106 (34.9%)	-	0	
	Non-sarcopenic	197 (65.1%)	-	0.5%	
			*p* = 0.023	*p* = 0.353	
Hu et al. [16]		153			12
	Sarcopenic	45 (29.4%)	91.1%	2.2%	
	Non-sarcopenic	108 (70.6%)	99.1%	0	
			*p* = 0.043	-	
Kobayashi et al. [4]		124			NA
	Sarcopenic	24 (19.3%)	-	-	
	Non-sarcopenic	100 (80.7%)	-	-	
			*p* = 0.343	*p* = 0.946	
Kroh et al. [25]		70			60
	Sarcopenic	33 (47.1%)	45%	3%	
	Non-sarcopenic	37 (52.9%)	13.6%	8%	
			*p* = 0.035	*p* = 0.616	
Lodewick et al. [26]		80	-	-	3
	Sarcopenic	31 (39%)	-	10.5%	
	Non-sarcopenic	49 (61%)	-	3.5%	
			-	-	
Peng et al. [27]		259	40%	0.8%	NA
	Sarcopenic	41 (16%)	-	-	
	Non-sarcopenic	218 (84%)	-	-	
			-	-	
Vledder et al. [28]		196	-	-	29
	Sarcopenic	38 (19%)	20%	42.9%	
	Non-sarcopenic	158 (81%)	49.9%	-	
			*p* < 0.001	-	
Wu et al. [29]		1172			NA
	Sarcopenic	421 (35.9%)	(Significantly worse)	-	
	Non-sarcopenic	751 (65.1%)	-	-	
			*p* < 0.001	-	
Yabusaki et al. [30]		195			37
	Sarcopenic	89 (45.6%)	85.3 months	2.2%	
	Non-sarcopenic	106 (54.4%)	96.3 months	2.8%	
			*p* = 0.72	*p* = 0.8	
Berardi et al. [7]		234	-	-	3
	Sarcopenic	91 (38.8%)	-	1.3%	
	Non-sarcopenic	85 (53.5.%)	-	0	
			-	-	
Kim et al. [32]		159	70.2%	27%	1
	Sarcopenic	74 (46.5%)	-	-	
	Non-sarcopenic	85 (53.5%)	-	-	
			-	-	
Hou et al. [33]		153	21.4%	71.2%	41.3
	Sarcopenic	77 (50.3%)	-	-	
	Non-sarcopenic	76 (49.7%)	-	-	
			-	-	
Zhou et al. [34]		195	-	79.1%	NA
	Sarcopenic	89 (45.6%)	21 months	-	
	Non-sarcopenic	106 (54.4%)	6 months	-	
			*p* < 0.001	-	
Liu et al. [5]		182	63%	-	32.5
	Sarcopenic	48 (26.4%)	-	-	
	Non-sarcopenic	134 (73.6%)	-	-	
			-	-	
Xiong et al. [36]		114	34.3%		60
	Sarcopenic	58 (50.8%)	-	-	
	Non-sarcopenic	56 (49.2%)	-	-	
			-	-	
Pessia et al. [37]		74			32
	Sarcopenic	48 (64.8%)	Significantly worse	-	
	Non-sarcopenic	26 (35.2%)	-	-	
			p = 0.0297	-	
Xu et al. [38]		1420			12
	Sarcopenic	458 (32.2%)	Significantly worse	-	
	Non-sarcopenic	962 (67.8%)	-	-	
			*p* = 0.002	-	
Furukawa et al. [8]		63			36
	Sarcopenic	33 (52.3%)	63.9%	-	
	Non-sarcopenic	30 (47.7%)	77.7%	-	
			*p* = 0.02	-	

**Table 6:** Odds ratio for right and left colon.

Studies	Patients	Right Colon	Left Colon	Odds Ratio	Lower 95% CI	Upper 95% CI
Bajric et al. (2022) [24]	355	180	175	4	3.2083	6.1028
Kobayashi et al. (2017) [4]	124	69	55	0.5555	0.2993	1.031
Lodewick et al. (2015) [26]	80	24		1.8452	0.9799	3.4748
Peng et al. (2011) [27]	259	191	68	0.8176	0.5317	1.2572
Runkel et al. (2021) [9]	94	60	34	1.751	0.9954	3.081
Vledder et al. (2012) [28]	196	116	80	0.8026	0.5121	1.2578
Berardi et al. (2020) [7]	234		96	1.5511	1.0884	2.2106
Wijk et al. (2021) [35]	128	58	70	2.6167	1.678	4.0806
Liu et al. (2020) [5]	182	82	100	1.0654	0.6918	1.6406
Pessia et al. (2021) [37]	74	34	40	2.6118	1.4566	4.6831
Furukawa et al. (2021) [8]	118	48	70	1.3345	0.7948	2.2407

## Data Availability

Not applicable.
